# Levels of asymmetric dimethylarginine in plasma and aqueous humor: a key risk factor for the severity of fibrovascular proliferation in proliferative diabetic retinopathy

**DOI:** 10.3389/fendo.2024.1364609

**Published:** 2024-06-12

**Authors:** Xinyang Guo, Wei Jin, Yiqiao Xing

**Affiliations:** Eye Center, Renmin Hospital of Wuhan University, Wuhan, Hubei, China

**Keywords:** diabetic retinopathy, fibrovascular proliferation, asymmetric dimethylarginine, targeted metabolomics, plasma, aqueous humor

## Abstract

**Introduction:**

Proliferative diabetic retinopathy (PDR) is a common diabetes complication, significantly impacting vision and quality of life. Previous studies have suggested a potential link between arginine pathway metabolites and diabetic retinopathy (DR). Connective tissue growth factor (CTGF) plays a role in the occurrence and development of fibrovascular proliferation (FVP) in PDR patients. However, the relationship between arginine pathway metabolites and FVP in PDR remains undefined. This study aimed to explore the correlation between four arginine pathway metabolites (arginine, asymmetric dimethylarginine[ADMA], ornithine, and citrulline) and the severity of FVP in PDR patients.

**Methods:**

In this study, plasma and aqueous humor samples were respectively collected from 30 patients with age-related cataracts without diabetes mellitus (DM) and from 85 PDR patients. The PDR patients were categorized as mild-to-moderate or severe based on the severity of fundal FVP. The study used Kruskal-Wallis test to compare arginine, ADMA, ornithine, and citrulline levels across three groups. Binary logistic regression identified risk factors for severe PDR. Spearman correlation analysis assessed associations between plasma and aqueous humor metabolite levels, and between ADMA and CTGF levels in aqueous humor among PDR patients.

**Results:**

ADMA levels in the aqueous humor were significantly greater in patients with severe PDR than in those with mild-to-moderate PDR(*P=*0.0004). However, the plasma and aqueous humor levels of arginine, ornithine, and citrulline did not significantly differ between mild-to-moderate PDR patients and severe PDR patients (*P>*0.05). Binary logistic regression analysis indicated that the plasma (*P=*0.01) and aqueous humor (*P=*0.006) ADMA levels in PDR patients were risk factors for severe PDR. Furthermore, significant correlations were found between plasma and aqueous humor ADMA levels (*r=*0.263, *P*=0.015) and between aqueous humor ADMA and CTGF levels (*r=*0.837, *P*<0.001).

**Conclusion:**

Elevated ADMA levels in plasma and aqueous humor positively correlate with the severity of FVP in PDR, indicating ADMA as a risk factor for severe PDR.

## 1 Introduction

With the global prevalence of diabetes on the rise, the incidence of diabetic retinopathy (DR) is also increasing (1). Recent research has revealed a greater incidence of DR in Asian countries than in Western countries (2). Among DR cases, vision-threatening diabetic retinopathy (VTDR) accounts for approximately 10% of cases (2). Proliferative diabetic retinopathy (PDR) is a significant category of VTDR and serves as a crucial cause of visual impairment and potential blindness in working-age diabetic patients. The prognosis of patients with PDR can vary due to the extent of fibrovascular proliferation (FVP) in the fundus. PDR with severe FVP often leads to retinal detachment, while PDR patients with simple vitreous hemorrhage generally have a better prognosis than those with retinal detachment due to severe FVP (3, 4). Moreover, young patients with PDR are more susceptible to severe FVP, resulting in a poorer visual prognosis (5). This condition significantly impacts their quality of life and ability to work, emphasizing the need for extensive attention and care.

Research has demonstrated that the degree of FVP in PDR patients is associated with factors such as renal dysfunction, age, duration of DR, hypertension, and smoking history (6). Furthermore, connective tissue growth factor (CTGF) plays a role in the progression of retinal fibrosis and is a positive correlation with the severity of fundus FVP in patients with DR (7). However, while these risk factors provide some insight, they fail to fully explain the underlying causes and molecular mechanisms involved in the varying degrees of FVP in PDR patients. Therefore, further investigations are necessary to explore the specific bioactive molecules responsible for promoting FVP in the eyes of PDR patients.

In recent years, metabolomics studies have revealed significant upregulation of arginine pathway metabolites in the plasma and aqueous humor of patients with DR, and these metabolites are correlated with the severity of DR (8–10). Research has shown that arginine metabolism promotes skeletal muscle and cardiac fibrosis in patients with muscular dystrophy (11).Additionally, arginine methylation plays a role in regulating epithelial-mesenchymal transition (12). Protein arginine methyltransferase has been shown to promote the progression of fibrosis in conditions such as diabetic nephropathy, idiopathic pulmonary fibrosis, and liver cirrhosis (13). However, the associations between arginine pathway metabolites and fundal FVP in patients with PDR have not been established. Therefore, this study aimed to investigate the correlation between specific arginine pathway metabolites (L-arginine, ADMA, L-ornithine, and L-citrulline) and the severity of FVP in PDR patients, providing valuable insights into the early warning of intraocular FVP in cases of severe PDR.

## 2 Methods

### 2.1 Ethical statement

The retrospective cross-sectional study involving human participants conducted in this research has undergone thorough review and approval by the Clinical Research Ethics Committee of Renmin Hospital of Wuhan University (WDRY2023-K083). This study strictly adhered to the principles outlined in the Declaration of Helsinki and followed international ethical guidelines, including the Measures for the Ethical Review of Biomedical Research Involving Humans, as well as relevant domestic laws and regulations. The patients provided their written informed consent to participate in this study.

### 2.2 Patient recruitment

**Figure 1:**
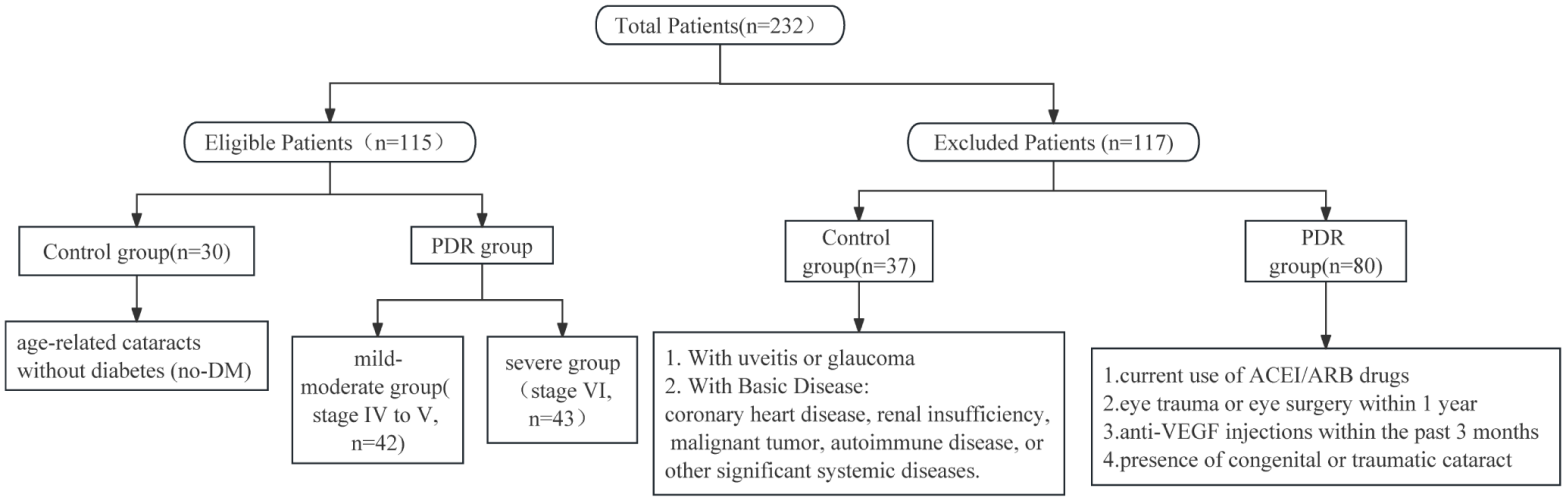
Patient inclusion and exclusion criteria.

This study randomly recruited a total of 232 patients, aged between 18 and 79 years, from the Eye Center of Renmin Hospital of Wuhan University, during the period from January to July 2023. The inclusion criteria for the PDR group involved meeting the diagnostic criteria for PDR according to the latest guidelines, and additionally, participants needed to exhibit varying degrees of fibrovascular proliferation in the fundus. Patients in the PDR group meeting any of the following criteria were excluded from this study: current use of angiotension converting enzyme inhibitors/angiotensin II receptor blockers (ACEI/ARB), a history of ocular trauma or eye surgery within the past year, or recent use of intravitreal anti-VEGF drug injections within the last 3 months (**Figure 1**). The inclusion criteria for the control group were defined as patients with age-related cataracts without concurrent diabetes mellitus (DM). Patients in the control group meeting any of the following criteria were excluded: congenital cataract, traumatic cataract, age-related cataract with uveitis or glaucoma, coronary heart disease, renal insufficiency, malignant tumor, autoimmune disease, and other serious systemic underlying diseases. A total of 115 patients were ultimately included in the study, comprising 85 patients in the PDR group and 30 patients in the control group. To clarify whether the sample size included in the study meets statistical requirements, we conducted a sample size analysis using PASS 2021. Based on preliminary results, the relevant abundance mean of aqueous humor ADMA in the control group was 1, while in the mild PDR group, it was 1.9 with a standard deviation of 0.82, and in the severe PDR group, it was 2.7 with a standard deviation of 0.82. With a two-sided α of 0.05 and a test power (1-β) of 90%, utilizing Kruskal-Wallis Tests (Simulate), we calculated that each group requires a minimum of 8 subjects, totaling at least 24 subjects. Clinical data collection included gender, age, blood pressure, complications, hemoglobin A1C (HbA1C), and relevant blood biochemistry indicators.

### 2.3 PDR severity grading

In alignment with the 2019 International Classification of DR by the American Academy of Ophthalmology (14), this study refined the stratification of proliferative diabetic retinopathy (PDR) stages based on the severity of fundal FVP. This was accomplished by referencing the DR grading standard established in 1984 by the Chinese Medical Association. DR was divided into six stages, and PDR corresponded to stages IV-V, which included the early proliferative stage (stage IV), fibroproliferative stage (stage V), and late proliferative stage (stage VI). Neovascularization of the retina (NVE) and neovascularization of the disc (NVD) occur in stage IV (early proliferative stage). Stage V (the fibroproliferative stage) is characterized by the presence of fibrous membranes, which may be accompanied by preretinal or vitreous hemorrhage. In stage VI (the late proliferative stage), traction retinal detachment with a fibrovascular membrane is observed. In this study, the severity of proliferation in PDR patients was evaluated through preoperative fundus ultra-wide-field fundus photography and intraoperative microscopic findings. Based on the aforementioned criteria, PDR patients were classified into two groups: the mild-to-moderate group comprising stages IV to V, and the severe group consisting of stage VI.

### 2.4 Sample collection

After informed consent was obtained from the patients, fasting whole-blood samples were collected from them using EDTA-containing anticoagulant tubes. The blood was immediately centrifuged at 4500 rpm and 4°C for 15 min, and the resulting supernatant was carefully divided into cryopreservation tubes. After the tubes were briefly exposed to liquid nitrogen for 5 min, they were stored at -80°C. During the surgery, approximately 150 μL of aqueous humor was collected from one eye in the PDR group. In the control group, approximately 150 μL of aqueous humor was collected from one eye with a cataract. The aqueous humor samples were then transferred to cryopreservation tubes and rapidly frozen in liquid nitrogen for 5 min before being stored at -80°C for preservation. The interval between the collection of plasma and aqueous humor sample collection was within 2 hours.

### 2.5 Target metabolite UPLC-MS/MS analysis

Two hundred microliters of acetonitrile containing the internal standard was added to 100 μL of plasma or aqueous humor. The mixture was vortexed for 30 seconds. The mixture was subsequently centrifuged at 13000 rpm for 15 min at 4°C, after which the supernatant was transferred to a clean tube and dried using a SpeedVac (Labconco, USA). The dried extract was redissolved in 80% acetonitrile and analyzed via ultra-performance liquid chromatography with tandem mass spectrometry (UPLC–MS/MS) conducted on a Waters Acquity UPLC-system coupled with a 5500 QTRAP system (SCIEX). Chromatographic separation was achieved on a Waters Acquity UPLC BEH Amide Column (2.1 mm× 100 mm, 1.7 μm, Waters) using a flow rate of 0.3 mL/min at 40°C during an 8 min gradient (0–0.5 min 20% A, 0.5–2 min 20%-80%A, 2–6.5 min 80%A, 6.5–8 min 80%-20%A) using buffer A (5 mM ammonium formate and 0.1% (v/v) formic acid in water) and buffer B (0.1% (v/v) formic acid in acetonitrile).

Mass spectrometry was performed in positive mode with an electrospray source voltage set to 5000 V. The analytes were monitored in multiple reaction monitoring (MRM) mode using precursor-to-product ion transitions of m/z 175.2 → 70.0 for arginine, m/z 203.2 → 70.0 for ADMA, m/z 176.1 → 159.0 for citrulline, m/z 133.1 → 70.2 for ornithine and m/z 185.1 → 75.0 for arginine-13C6 and 15N4 (IS). The collision energies were 30.0 eV for arginine, 40 eV for ADMA, 15 eV for citrulline, 15 eV for ornithine, and 33.5 eV for IS. Peak determination and area integration were performed using Analyst 1.7.1 (SCIEX) and SCIEX OS1.4.0 software (SCIEX).

### 2.6 ELISA for CTGF analysis

Detection of CTGF was performed using human-specific ELISA kits (UpingBio, SYP-H0225). In the experiment, wells were designated for standard samples, sample dilution, blanks, and test samples. Standard wells received 50μL of standards at different concentrations, sample dilution wells received 50μL of sample dilution fluids, blank wells remained empty, and test sample wells received 50μL of the samples to be tested. Subsequently, 100μL of biotinylated antibody was added to each well and incubated at 37°C for 60 minutes. After washing the plate and patting it dry on absorbent paper, this process was repeated five times. Next, each well received 100μL of HRP-conjugated avidin and was incubated at 37°C for 20 minutes. After another five washes, all wells were loaded with 100μL of substrate mixture and incubated at 37°C for 15 minutes. Finally, 50μL of stop solution was added to each well, and the absorbance (OD values) of each well was read at 450nm wavelength using an ELISA reader.

### 2.7 Statistical analysis

The statistical analysis was conducted using SPSS 27.0 software. The normality of the measurement data was assessed using the Shapiro Wilk test. For normally distributed data, the mean ± standard deviation was used for representation, and the independent sample t test was used for comparing two groups. Non-normally distributed data are represented herein as medians and quartiles. Comparisons between two groups were conducted using the Mann-Whitney U test, while comparisons between three groups were performed using the Kruskal-Wallis test. Categorical data were analyzed using the chi-square test or continuity-corrected chi-square test. Multivariable binary logistic regression was employed for the analysis of risk factors. The correlation analysis was performed using Spearman correlation tests. Using GraphPad Prism 9.0 mapping software, outliers were identified as data points with relevant abundance values outside the 5–95th percentile of metabolite quantification.

## 3 Results

### 3.1 Demographic data

This study involved a total of 115 subjects, with 85 individuals in the PDR group and 30 in the group of age-related cataract patients without a history of diabetes. The number and reasons for excluded patients are shown in **Figure 1**. **Table 1** presents the clinical data of these subjects. The analysis revealed no significant differences in terms of sex (*P=*0.076), blood pressure (SBP, *P=*0.238; DBP, *P=*0.463), serum creatinine levels (*P=*0.102), or lipid levels(TCh, *P=*0.824; TG, *P=*0.319) between the PDR group and the control group. The age of the individuals in the control group was significantly greater than that of the individuals in the PDR group (*P* < 0.001).

**Table 1:** Demographic data between PDR patients and nondiabetic patients.

Variables	Non-diabetes	PDR patients	P-Value
Sex (% male)	46.67	51.76	0.076
Age	63.80 ± 8.23	52.94 ± 9.47	<0.001**
SBP (mmHg)	132 ± 18	137 ± 20	0.238
DBP (mmHg)	71 ± 10	72 ± 10	0.463
Cr (μmol/L)	66 (52,78)	71(62,89)	0.102
TCh(mmol/L)	4.60 ± 1.11	4.65 ± 0.92	0.824
TG(mmol/L)	1.27 ± 0.38	1.38 ± 0.57	0.319

The study groups were compared based on their demographics and comorbidities. To compare the differences between the two groups, A two-sample t tests were used for age, SBP, DBP, TCh, and TG, with the means and standard deviations presented. The Wilcoxon rank sum test was performed for creatinine levels, with the median and interquartile range presented. The chi-squared test was used to compare sex differences. SBP, systolic blood pressure; DBP, diastolic blood pressure; Cr, creatinine; TCh, total cholesterol; TG, triglyceride, ** represents *P<*0.01.

The PDR group was categorized based on the severity of FVP into two subgroups: mild-to-moderate PDR (42 patients) and severe PDR (43 patients). **Table 2** presents the clinical data of these subjects. Age (*P=*0.333), sex (*P=*0.829), HbA1c levels (*P=*0.512), and history of hypertension (*P=*0.229) or diabetic nephropathy (DN) (*P=*0.052) did not significantly differ between the mild-to-moderate PDR and severe PDR groups. However, significant differences were observed in the duration of diabetes (P=0.003) and serum creatinine levels (P=0.032). The mild-to-moderate PDR group had a longer duration of diabetes and lower serum creatinine levels than did the severe PDR group. This implies a potential correlation between the duration of diabetes and the severity of PDR. Furthermore, the significant difference in creatinine levels indicates that patients in the severe PDR group exhibit a decline in kidney function compared to those in the mild to moderate PDR group.

**Table 2:** Demographic data between patients with mild-to-moderate PDR and patients with severe PDR.

Variables	Mild-to-Moderate Diabetes	Severe Diabetes	P Value
Sex (% male)	52.38	53.49	0.829
Age	53.95 ± 9.93	51.95 ± 9.00	0.333
HbA1c (%)	8.01 ± 1.91	8.28 ± 1.76	0.512
DM Duration (years)	12.93 ± 3.142	10.37 ± 4.39	0.003**
Cr	70(62,73)	73(67,92)	0.032*
HTN	40.48%	53.49%	0.229
DN	2.38%	13.95%	0.052

The DR group was divided into mild-to-moderate and severe subgroups based on the severity of PDR. The two subgroups were compared in terms of their demographics and comorbidities. A two-sample t test was used to compare the differences between the subgroups in terms of age, HbA1c level, and duration of diabetes, and the means and standard deviations are presented. The Wilcoxon rank sum test was used to compare the differences in creatinine levels between the two subgroups, and the medians and interquartile ranges are presented. The chi-square test was used to compare sex and comorbidities. HbA1c, glycated hemoglobin A1c; Cr, creatinine; HTN, hypertension; DN, diabetic nephropathy. * represents *P<*0.05, ** represents *P<*0.01.

### 3.2 Comparison of the levels of four metabolites

**Figure 2:**
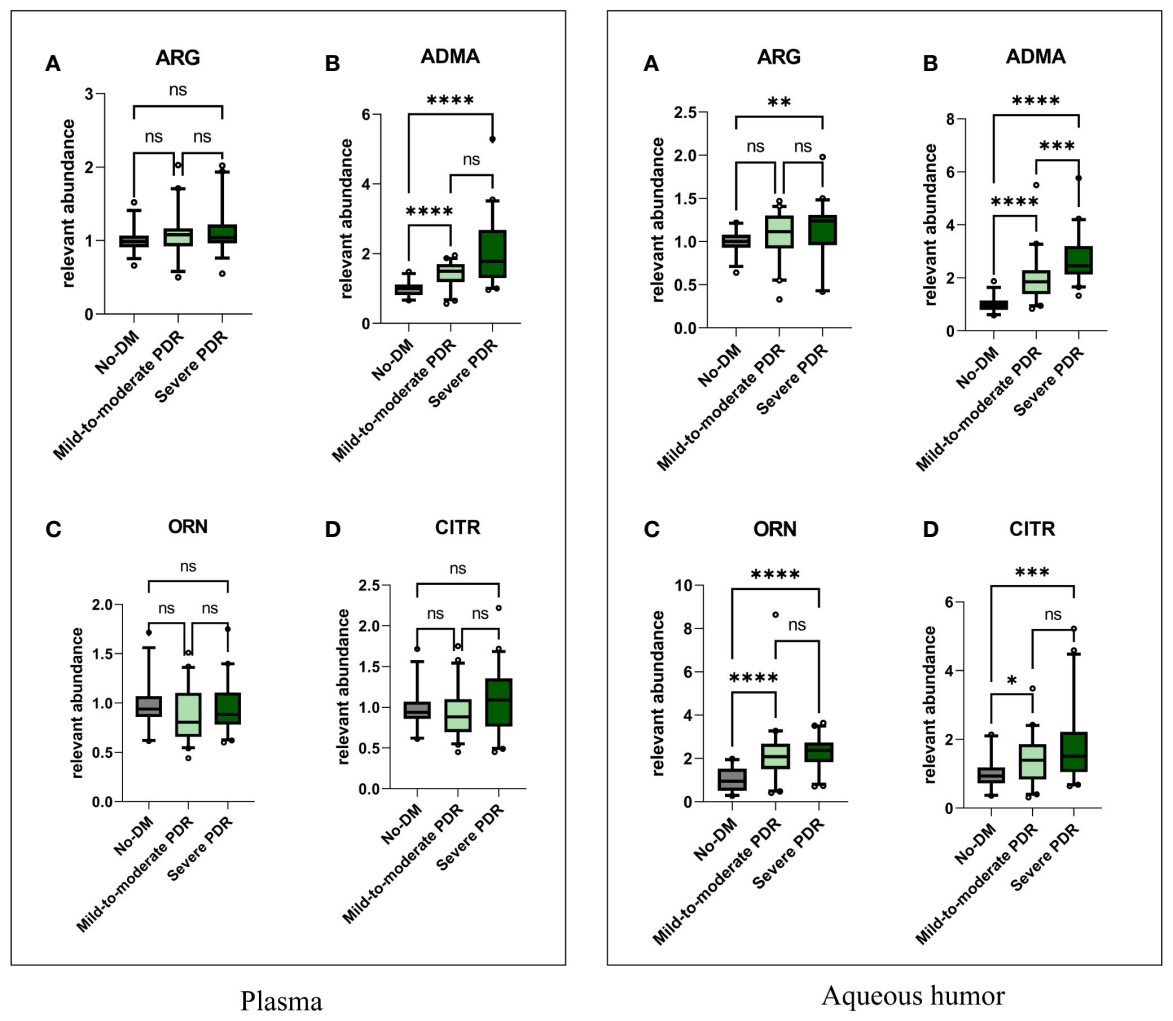
The comparisons of plasma and aqueous humor metabolite levels. In the plasma, Figures **(A–D)** respectively represent the comparison of plasma levels of arginine, ADMA, ornithine, and citrulline among the three groups. In the aqueous humor, Figures **(A–D)** respectively represent the comparison of aqueous humor levels of arginine, ADMA, ornithine, and citrulline among the three groups. Comparisons between groups were performed using the Kruskal Wallis test. No-DM represents nondiabetic patients. * represents P<0.05, ** represents P<0.01,*** represents P<0.001, and **** represents P<0.0001, ns represents P>0.05.

To investigate the differences in these four metabolites among nondiabetic individuals and those with varying severities of PDR, we analyzed the metabolite levels in three distinct patient groups (**Figure 2**). The plasma levels of ADMA in the mild-to-moderate and severe PDR groups were both significantly greater than those in the nondiabetic group (*P<*0.001). The levels of arginine, ADMA, ornithine, and citrulline in the aqueous humor of the severe PDR group were significantly greater than those in the nondiabetic group (*P=*0.0086, *P<*0.0001; *P*<0.0001; *P=*0.0002). Notably, only the ADMA level in the aqueous humor differed between the mild-to-moderate PDR group and the severe PDR group, with the ADMA level in the severe PDR group being significantly greater than that in the mild-to-moderate PDR group (*P=*0.0004). However, no significant differences were observed in the levels of arginine, ornithine, or citrulline in the aqueous humor between the mild-to-moderate PDR group and the severe PDR group (*P>* 0.05).

### 3.3 Assessment of the correlation of metabolite levels between plasma and aqueous humor in the PDR group

**Figure 3:**
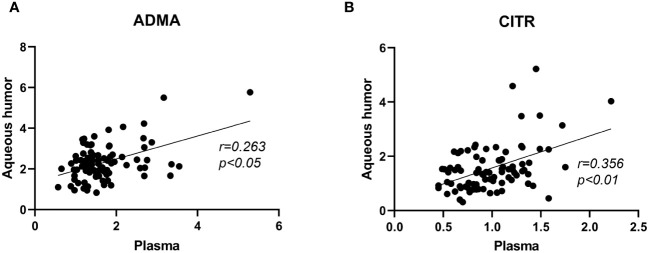
Correlation analysis of ADMA and citrulline levels between plasma and aqueous humor samples in PDR patients. Figure **(A)** represents the correlation analysis of ADMA levels between plasma and aqueous humor. Figure **(B)** represents the correlation analysis of citrulline levels between plasma and aqueous humor.The correlation analysis was performed using Spearman correlation tests.

To explore whether the trends in metabolite levels in aqueous humor and plasma are consistent, this study assessed the correlation of the levels of these four metabolites between plasma and aqueous humor (**Figure 3**). Correlation analysis revealed significant correlations between plasma ADMA levels and aqueous humor (*r*= 0.263, P < 0.015) and between plasma citrulline levels and aqueous humor (*r*= 0.356, P < 0.001). However, we did not observe a significant correlation between the levels of arginine (P = 0.285) in plasma or aqueous humor or between the levels of ornithine (P= 0.162) in plasma and aqueous humor (**Supplementary Figure 1**). Additionally, a significant correlation was found between ADMA and citrulline levels in the plasma (*r=*0.469, *P<*0.001) and in the aqueous humor (*r*= 0.475, P < 0.001; **Supplementary Figure 2**).

### 3.4 Binary logistic regression analysis

To further assess whether ADMA levels serve as risk factors for the severity of FVP in PDR patients, we conducted a multivariable binary logistic regression analysis while adjusting for confounding factors such as diabetes duration, HbA1c level, and serum creatinine value (as presented in **Table 3**). The odds ratio (OR) for plasma ADMA levels was 4.139, with a 95% confidence interval (CI) of 1.402 to 12.214 (*P=*0.010), and the OR for aqueous humor ADMA levels was 3.165, with a 95% CI of 1.402 to 7.148 (*P=*0.006).

**Table 3:** Logistic regression analysis of mild-to-moderate versus severe PDR.

Variables	OR	CI	P-Value
Plasma-ADMA	4.139	1.402–12.214	0.010*
Aqueous humor-ADMA	3.165	1.402–7.148	0.006**

Binary logistic regression analysis was performed with severe PDR as the outcome. The odds ratio is per 1 unit increase in plasma or aqueous humor. * represents *P<*0.05, ** represents *P<*0.01.

### 3.5 The relationship between the ADMA levels and connective tissue growth factor in the aqueous humor

**Figure 4:**
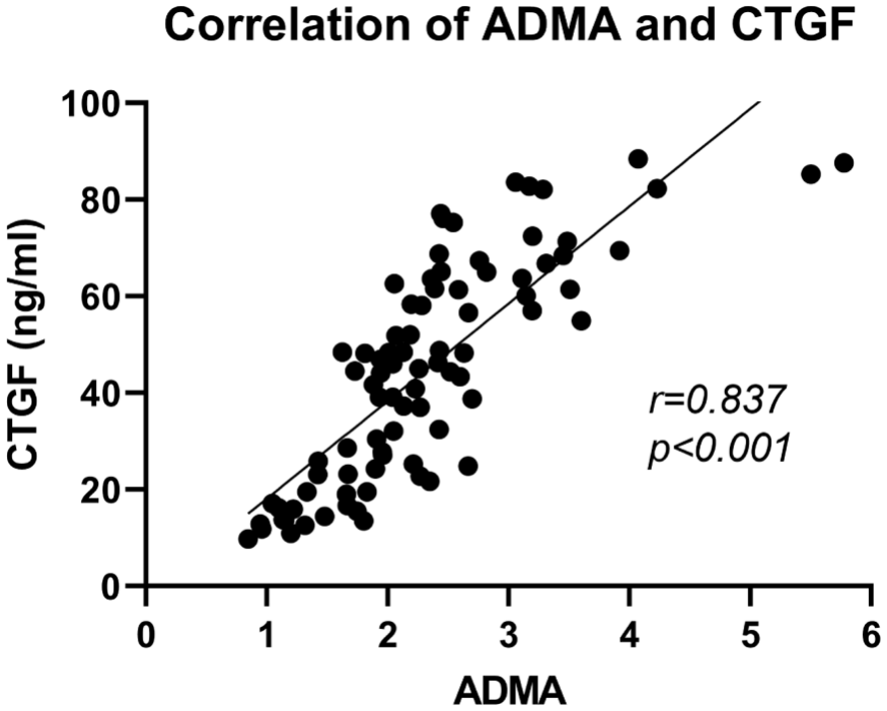
Correlation analysis of ADMA and CTGF in the aqueous humor among PDR patients. The correlation analysis was performed using Spearman correlation tests.

To further explore the connection between ADMA and the extent of fibrosis in PDR, we conducted a detailed analysis of the correlation between ADMA levels and CTGF in the aqueous humor (**Figure 4**). Our research findings revealed a positive correlation between the levels of ADMA and CTGF in the aqueous humor (*r=*0.837, *P<*0.01).

## 4 Discussion

This study aims to investigate the correlation between four arginine pathway metabolites (arginine, ADMA, ornithine, and citrulline) and the severity of FVP in PDR patients. Initially, we analyzed the differences in these metabolites among PDR patients with varying degrees of FVP. Significant differences were observed in the levels of ADMA in the aqueous humor among PDR patients with different severity levels of FVP. Subsequently, we observed a consistent trend in the changes of ADMA levels in both plasma and aqueous humor. Through further analysis, we confirmed ADMA as a risk factor for FVP in PDR patients. Finally, we also found a significant correlation between ADMA in the aqueous humor and CTGF, further indicating the crucial role of ADMA in the occurrence and development of FVP in PDR patients.

In recent years, there has been a notable increase in the occurrence and prevalence of type 2 diabetes mellitus among young individuals (15). Studies suggest that early-onset Type 2 diabetes patients are more likely to develop PDR compared to those with late-onset diabetes (16, 17). A study from Beixinjing found that the incidence of DR significantly increases in patients with diabetes duration of less than 10 years, while it decreases in those with a duration of over 10 years (18). Additionally, younger PDR patients are at higher risk of severe FVP compared to older patients (5). This indicates that individuals diagnosed with diabetes at a younger age are at a higher risk of rapidly developing severe PDR. In this study, we observed that the duration of diabetes in patients with mild-to-moderate PDR was significantly longer than in those with severe PDR. This phenomenon may be attributed to the younger age of onset of diabetes among some PDR patients in this study, leading them to progress to severe PDR within a short period of time. Therefore, early diagnosis and management of diabetes are crucial for preventing the occurrence of severe PDR.

In this study, we utilized age-related cataract patients as the baseline control group and employed their metabolic levels as a reference for the relative quantification of metabolites in the PDR group. However, we observed that age-related cataract patients typically tend to be older compared to PDR patients in clinical settings. Therefore, there is a significant age difference between the control and PDR groups in this study.

Our study observed differences in arginine metabolite levels between the control and PDR groups, which may be age-related. However, our study primarily focused on differences in metabolite levels among PDR patients with varying degrees of severity. There was no statistically significant difference in age between the mild-to-moderate and the severe PDR group. This indicates that age did not influence the comparison of metabolite levels between the mild-to-moderate and severe PDR groups.

In this study, we found that there was no statistically significant difference in ADMA levels in plasma samples between patients with mild-moderate and severe PDR. However, in aqueous humor samples, the ADMA levels in severe PDR patients were significantly higher than those in mild-moderate PDR patients. This suggests that the association between ADMA levels and the severity of FVP in PDR patients is more closely linked in the aqueous humor compared to plasma. This may be attributed to the aqueous humor being an intraocular fluid, providing a more sensitive reflection of metabolic changes within the eye.

Although our results indicate that ADMA levels in plasma do not significantly differ between mild-to-moderate and severe PDR patients, our correlation analysis between aqueous humor and plasma reveals a significant correlation in ADMA levels between these two compartments (**Figure 3**). This suggests a consistent trend in the changes of ADMA levels in both aqueous humor and plasma, potentially related to aqueous humor circulation. Aqueous humor is generated by the ciliary process and flows into the anterior chamber through the pupil. Subsequently, it traverses the trabecular meshwork, enters the scleral venous system, and eventually enters the systemic circulation (19). The aqueous humor circulation process serves as a pathway for the exchange of substances between intraocular and systemic circulation. Therefore, ADMA within the eye can enter the systemic circulation through the aqueous humor, leading to corresponding changes in ADMA levels in the plasma. Monitoring ADMA levels in the plasma can, to some extent, reflect changes in intraocular ADMA levels.

The ADMA level in the aqueous humor is elevated in severe PDR patients compared to those with mild-to-moderate PDR, suggesting a strong association between ADMA and FVP in PDR patients. Considering the correlation between metabolite levels and patients’ renal function (20), we incorporated blood creatinine levels and other potential confounding factors, including diabetes duration and HbA1c, into our multivariate binary logistic regression analysis. The results reveal that elevated plasma and aqueous humor ADMA levels were identified as risk factors for FVP in PDR patients. As the level of ADMA increased, the risk of severe FVP in PDR patients also increased. This implies a positive correlation between ADMA levels and the severity of FVP in PDR patients.

ADMA, an endogenous inhibitor of nitric oxide synthase (NOS), plays a crucial role in inhibiting the production of nitric oxide (NO) (21). In a high glucose environment, the production of ADMA increases while its degradation decreases (22). Elevated ADMA levels, by competitively binding with L-arginine for the active site of NOS, can impede the synthesis of NO, leading to endothelial dysfunction and tissue ischemia and hypoxia (23–25). Tissue hypoxia triggers the upregulation of hypoxia-inducible factor (HIF), activating multiple fibrosis signaling pathways (26). Furthermore, ADMA can induce epithelial/endothelial cell-mesenchymal transition (EMT/EndMT), resulting in the increased generation of myofibroblasts (27). A previous study has also revealed a correlation between the polymorphism of the protein arginine methyltransferase (PRMT1) gene and an increased incidence of PDR (28). PRMT1 is a crucial enzyme involved in ADMA synthesis, highlighting the close association between elevated ADMA levels and PDR. The severity of PDR is closely associated with the FVP in the fundus. Patients with severe PDR typically exhibit a substantial amount of FVP in the fundus. The fibroproliferative membrane formed by FVP can generate tension, further stretching the retina and inducing retinal detachment. ADMA plays a significant role in fostering tissue fibrosis. This may explain why ADMA functions as a risk factor for the severity of FVP in PDR. Further studies are needed to validate and explore the underlying molecular mechanisms involved.

These findings are consistent with the results of previous studies, such as that conducted by Abhary et al., which also reported elevated plasma ADMA levels among individuals with severe DR (29). Similarly, Peters et al. discovered that the plasma ADMA level was notably elevated in PDR patients compared to that in NPDR patients (30). Moreover, previous research has shown that levels of ADMA in the aqueous humor of individuals with severe PDR are significantly higher than those in individuals without diabetes (31). This suggests a crucial role for ADMA in both the occurrence and progression of DR. Nonetheless, earlier research has not established a significant correlation between ADMA levels in plasma and aqueous humor, which might be attributed to variations in detection techniques (31). In our study, we employed UPLC-MS/MS to increase the sensitivity of detection (32). Building on prior research, our study revealed the association between ADMA and the severity of FVP in PDR patients. This provides new insights into the early warning of intraocular FVP in cases of severe PDR.

CTGF plays an important role in basement membrane thickening, extracellular matrix synthesis, angiogenesis, and fibrosis and is emerging as a crucial biomarker for assessing the extent of ocular fundus fibrosis (33, 34). Our research revealed a significant correlation between the levels of ADMA and CTGF in the aqueous humor. These findings imply that ADMA may cooperate with CTGF or induce the expression of CTGF to promote the formation and development of FVP in PDR. Wang et al. found in their study that ADMA can induce the upregulation of transforming growth factor β (TGF-β), promoting fibrosis in renal glomerular endothelial cells (35). CTGF is a downstream effector molecule of TGF-β, where TGF-β can induce increased expression of CTGF, thereby promoting fibrosis (36). Therefore, ADMA may induce fibrosis in retinal tissue by upregulating CTGF through the TGF-β pathway. Further research is essential to elucidate the specific mechanisms underlying the involvement of ADMA in the FVP of PDR patients.

Previous research indicates that factors such as renal function, and the use of ACE inhibitors may influence the levels of ADMA and CTGF in plasma (20, 37–39). Patients with poorer renal function levels tend to have higher plasma levels of ADMA and CTGF (37, 39). In this study, there was a significant correlation between ADMA and CTGF levels in the aqueous humor of PDR patients. Furthermore, we found a significant correlation between the levels of metabolites in the aqueous humor and plasma. This suggests that renal function may serve as a potential confounding factor influencing ADMA and CTGF levels in the aqueous humor. Further research is necessary to validate this hypothesis.

However, several limitations should be considered in this study. First, the cross-sectional design utilized in this study limits our ability to determine the dynamic changes in metabolite levels and their temporal relationship with the progression of PDR. Employing longitudinal study designs would provide a better understanding of the association between metabolite alterations and disease progression. Secondly, although we observed a significant correlation between ADMA and CTGF levels, further basic experimental and molecular mechanism studies are still needed to elucidate the exact relationship between them and their interaction in the formation and development of FVP in PDR patients.

In conclusion, despite some limitations, this study provides new insights into the warning of intraocular FVP in cases of severe PDR. The study reveals a positive correlation between ADMA levels and the severity of FVP in PDR patients in both plasma and aqueous humor, indicating that ADMA is a risk factor for FVP in PDR patients. Future research can build upon this study by conducting animal or cell model experiments to assess the impact of modulating ADMA levels on PDR development. This will further elucidate the underlying mechanisms, thereby offering theoretical foundations for the prevention and treatment of FVP in PDR patients.

## Data availability statement

The raw data supporting the conclusions of this article will be made available by the authors, without undue reservation.

## Ethics statement

The studies involving human participants were reviewed and approved by the Clinical Research Ethics Committee of Renmin Hospital of Wuhan University (WDRY2023-K083). The patients provided their written informed consent to participate in this study.

## Author contributions

XG: Writing – original draft, Writing – review & editing. WJ: Conceptualization, Funding acquisition, Methodology, Project administration, Writing – review & editing. YX: Resources, Supervision, Validation, Visualization, Writing – review & editing.
