# Supplementary material for: Levels of asymmetric dimethylarginine in plasma and aqueous humor: a key risk factor for the severity of fibrovascular proliferation in proliferative diabetic retinopathy
Source: Front Endocrinol (Lausanne). 2024 Jun 12;15:1364609. doi: 10.3389/fendo.2024.1364609 (PMC11200173; doi:10.3389/fendo.2024.1364609)
Supplement: Supplementary file 1 [file DataSheet_1.docx]

Supplementary Material

# Supplementary Figures

##
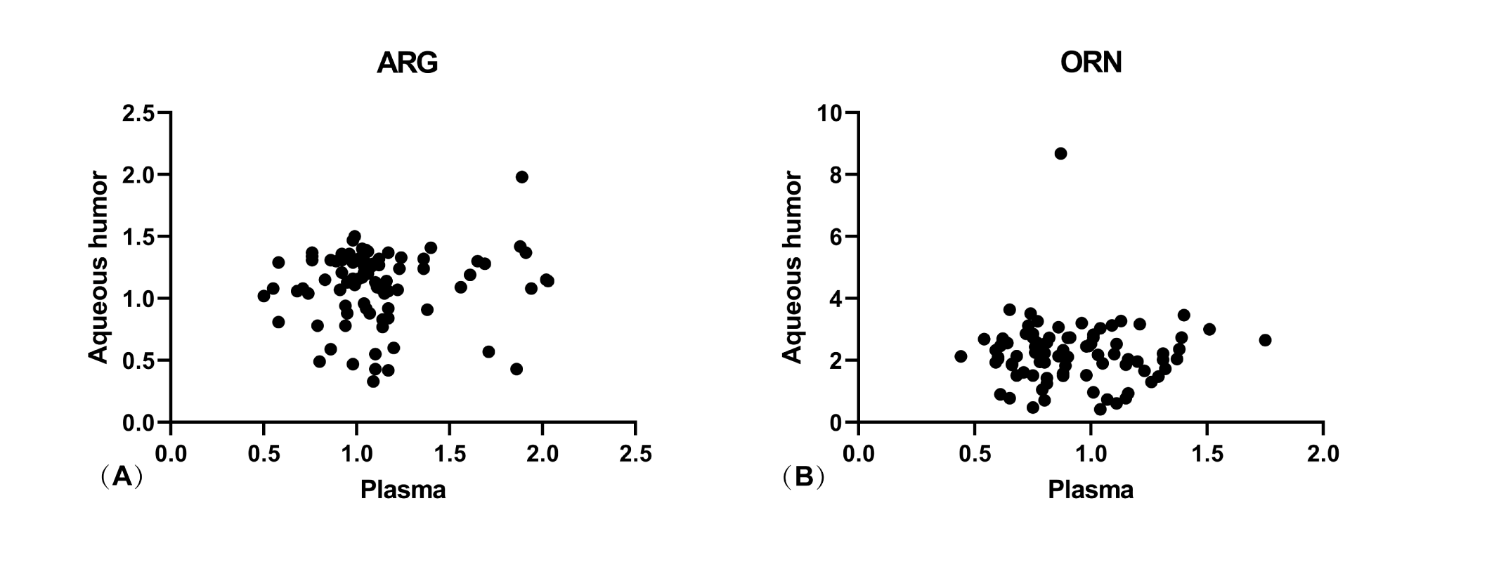
Supplementary Figure 1

**Supplementary Figure 1.** Correlation analysis of arginine and ornithine between plasma and aqueous humor. The correlation analysis was performed using Spearman correlation tests.

##
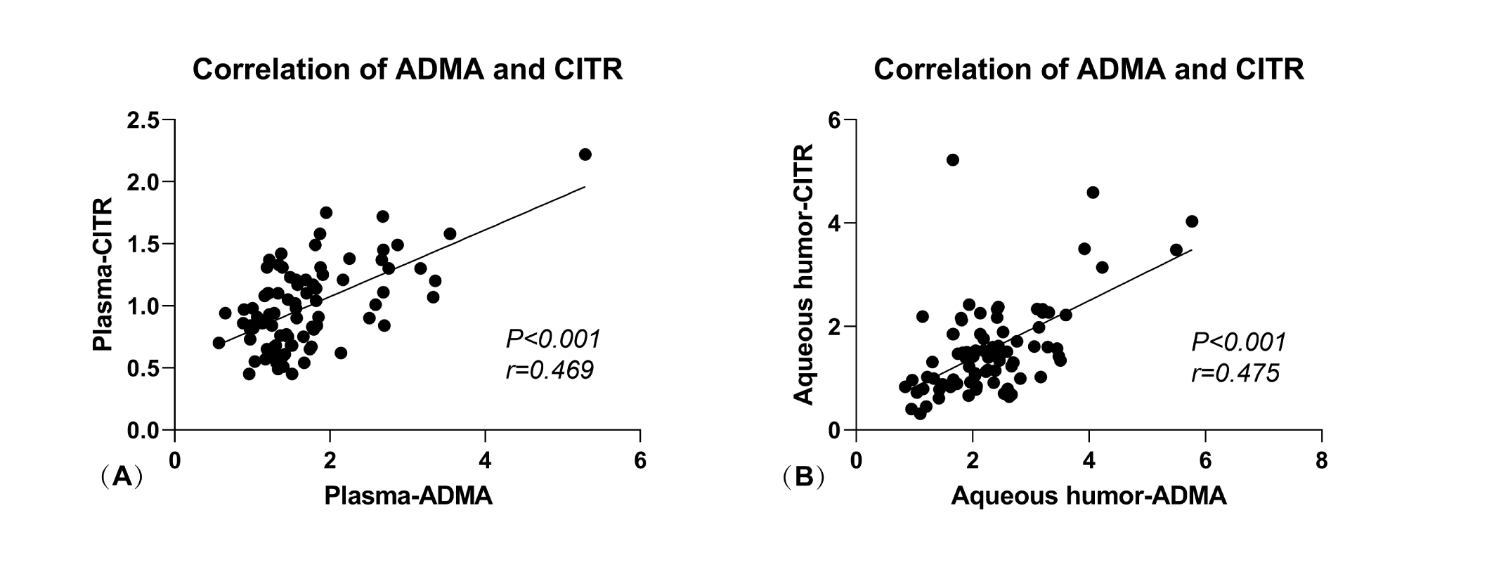
Supplementary Figure 2

Supplement 2 Correlation analysis between ADMA and citrulline. CITR represents citrulline. The correlation analysis was performed using Spearman correlation tests.
